# When a Pulmonary Embolism Precedes the Fall: Anticoagulation Challenges in Traumatic Intracranial Hemorrhage

**DOI:** 10.7759/cureus.101974

**Published:** 2026-01-21

**Authors:** Summiya Nasim, Rizwan Mushtaq, Kamran Mushtaq

**Affiliations:** 1 Internal Medicine, Parkview Health, Fort Wayne, USA; 2 Medicine, Ayub Medical College, Abbottabad, PAK; 3 Internal Medicine, Northeast Internal Medicine Associates, LaGrange, USA

**Keywords:** anticoagulation, direct oral anticoagulant, intracranial hemorrhage, mechanical thrombectomy, multidisciplinary care, pulmonary embolism, saddle pulmonary embolism, traumatic brain injury

## Abstract

Pulmonary embolism (PE) is typically managed with urgent systemic anticoagulation or reperfusion therapy. However, management becomes complex when PE occurs concurrently with traumatic intracranial hemorrhage (ICH), where anticoagulation may worsen bleeding.

A 74-year-old male patient presented after syncope and traumatic head injury. He was hypoxic and tachycardic on arrival with concern for right ventricular strain. Given persistent clinical concern, CT angiography of the chest demonstrated a massive saddle PE. CT of the head revealed traumatic subarachnoid hemorrhage and a small subdural hematoma with an occipital skull fracture, with no midline shift/mass effect, and an admission Glasgow Coma Scale (GCS) of 15/15. He underwent urgent mechanical thrombectomy with inferior vena cava (IVC) filter placement. A cautious heparin infusion was initiated without a bolus following neurosurgical approval and close neurological monitoring. Early repeat CT demonstrated interval hemorrhage progression, but subsequent imaging showed stability. His activated partial thromboplastin time (aPTT) was titrated carefully without clinical deterioration, and he was transitioned to apixaban without a loading dose. He remained neurologically stable and improved clinically prior to discharge.

This case illustrates the competing priorities of preventing fatal thromboembolism while minimizing hemorrhagic progression in traumatic ICH. Mechanical thrombectomy, serial imaging, and staged anticoagulation guided by multidisciplinary collaboration allowed safe re-initiation of anticoagulation.

## Introduction

Pulmonary embolism (PE) remains a major global cause of morbidity and mortality and requires rapid diagnosis and treatment. Standard therapy includes systemic anticoagulation and, in high-risk cases, reperfusion strategies such as catheter-directed or surgical thrombectomy [1].

When PE occurs concurrently with traumatic intracranial hemorrhage (ICH), clinical decision-making becomes exceptionally challenging. Anticoagulation is the cornerstone of PE management, yet it may worsen active hemorrhage, while delays in therapy increase the risk of right ventricular failure and death [2]. Because existing guidelines provide limited direction for patients presenting with both life-threatening conditions simultaneously, management must be individualized and strongly multidisciplinary.

We describe a case of traumatic ICH with saddle PE requiring urgent thrombectomy and carefully timed anticoagulation resumption. This case highlights practical considerations in balancing thrombotic and hemorrhagic risks, the value of serial neuroimaging, and the role of coordinated care between neurosurgery, hematology, critical care, and interventional teams.

## Case presentation

**Figure 1:**
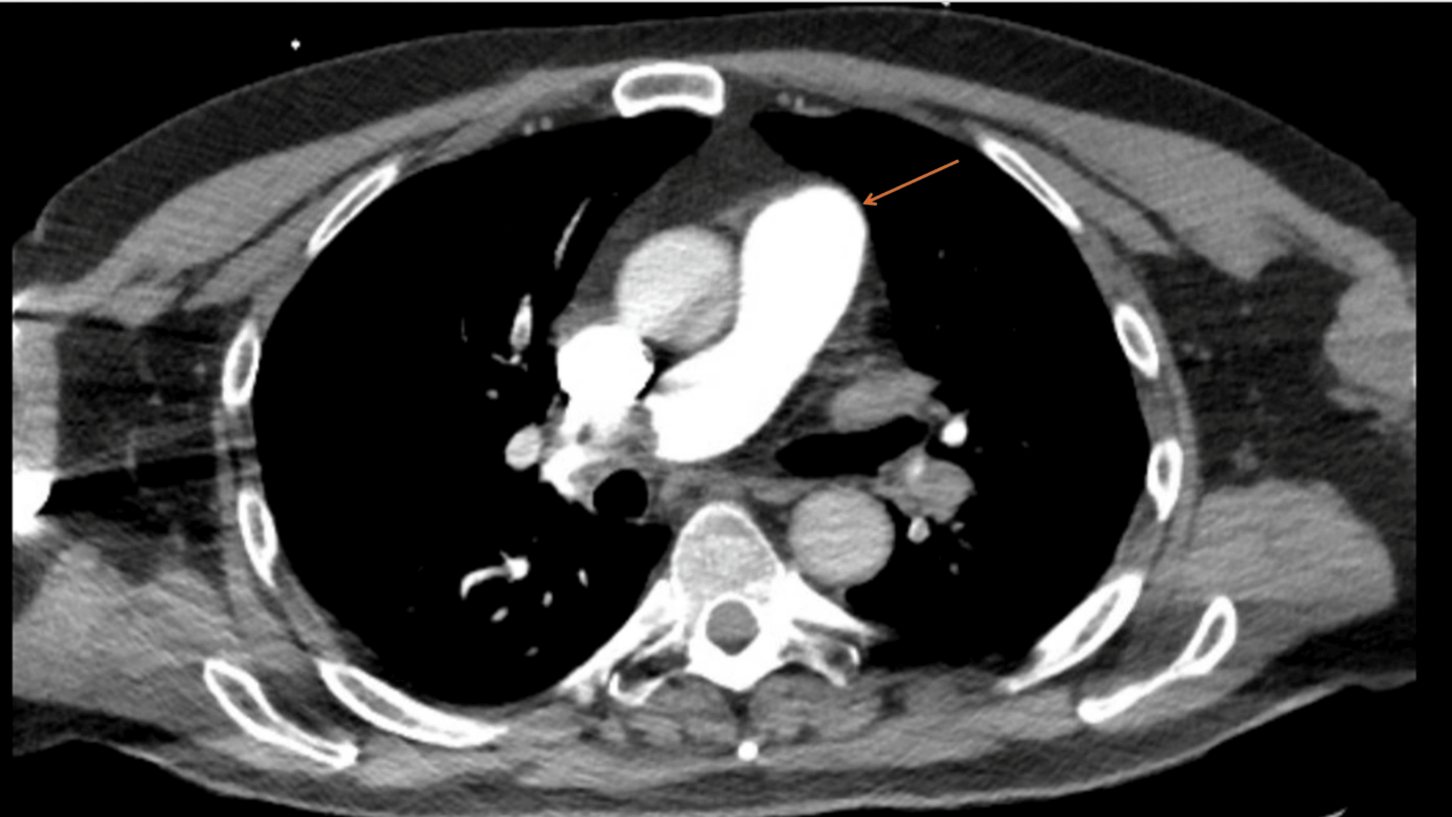
CT angiography of the chest demonstrating saddle pulmonary embolism (arrow) The arrow indicates a thrombus within the main pulmonary artery consistent with a saddle pulmonary embolism.

A 74-year-old male patient with a history of ulcerative colitis and prior venous thromboembolism presented to the emergency department after syncope with a ground-level fall. He was not on any anticoagulant or antiplatelet medication at the time of admission. On arrival, he was hypoxic and tachycardic but remained normotensive without evidence of shock. CT angiography of the chest demonstrated a large saddle PE extending into the main pulmonary arteries with evidence of right ventricular strain (Figure 1).

**Figure 2:**
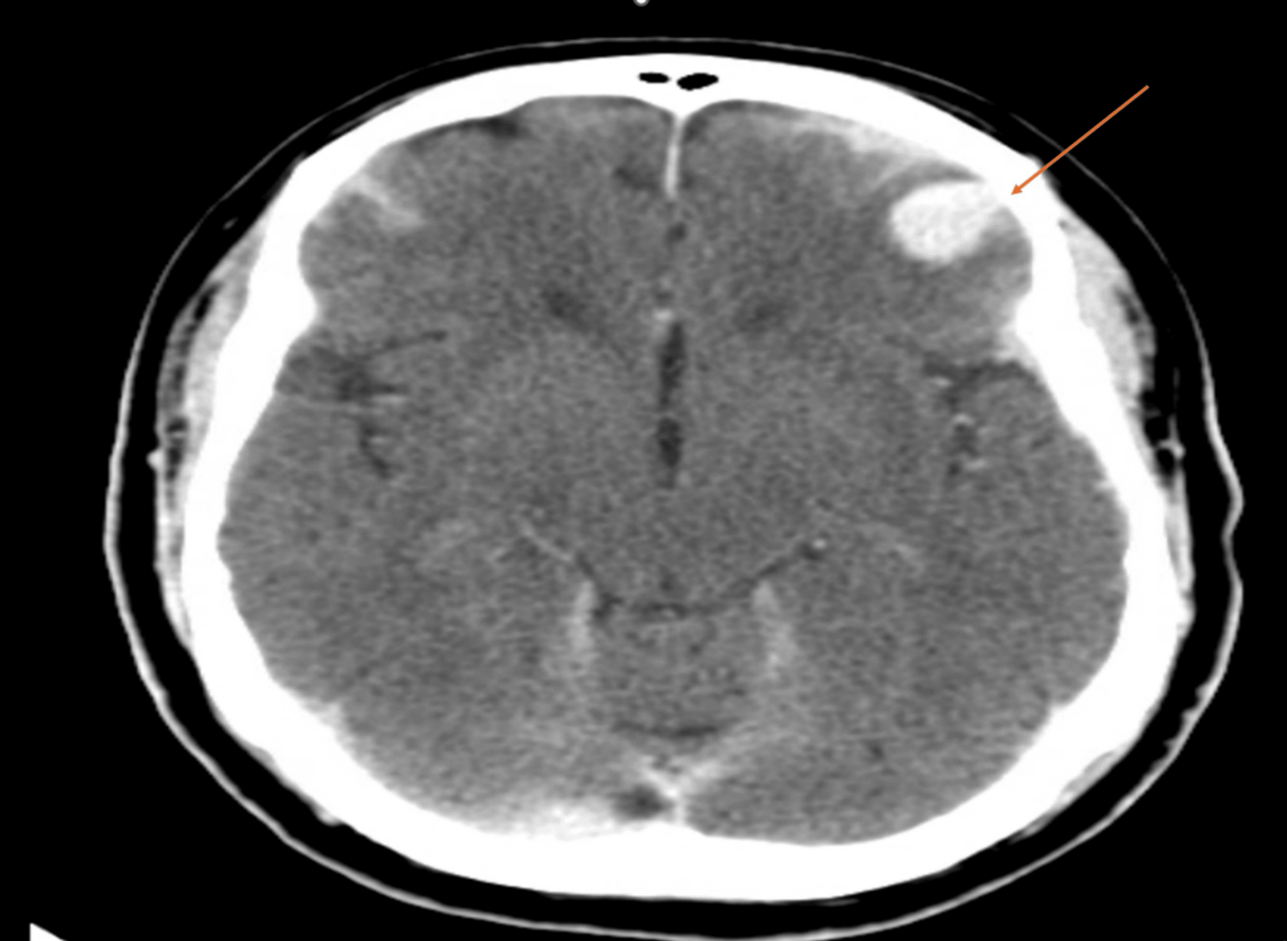
Initial CT of the head showing traumatic hemorrhage (arrow) Arrow highlights acute traumatic intracranial hemorrhage involving subarachnoid and subdural components.

Initial non-contrast CT of the head demonstrated traumatic subarachnoid hemorrhage, a bifrontal acute subdural hemorrhage measuring approximately 2 mm in thickness, and an occipital skull fracture (Figure 2), with associated frontal cerebral contusion. This initial CT of the head was obtained on hospital day 0 (presentation). His admission Glasgow Coma Scale (GCS) was 15/15. A consultant neurosurgeon recommended conservative management with serial neurological examinations and repeat imaging. 

Given significant clot burden and hemodynamic concern, the patient underwent catheter-directed mechanical thrombectomy by interventional radiology with successful thrombus retrieval, followed by inferior vena cava (IVC) filter placement on hospital day 0. Given the life-threatening nature of the massive PE, neurosurgery recommended proceeding with thrombectomy using low-dose heparin during the procedure to prevent catheter thrombosis, with a plan for a repeat non-contrast CT head post procedure. Pre-procedure CT angiography demonstrated a large saddle PE, and post-procedural imaging/clinical improvement supported effective reduction in thrombus burden. A low-intensity intravenous unfractionated heparin infusion was initiated without bolus after neurosurgical clearance, with careful activated partial thromboplastin time (aPTT)-guided titration.

**Figure 3:**
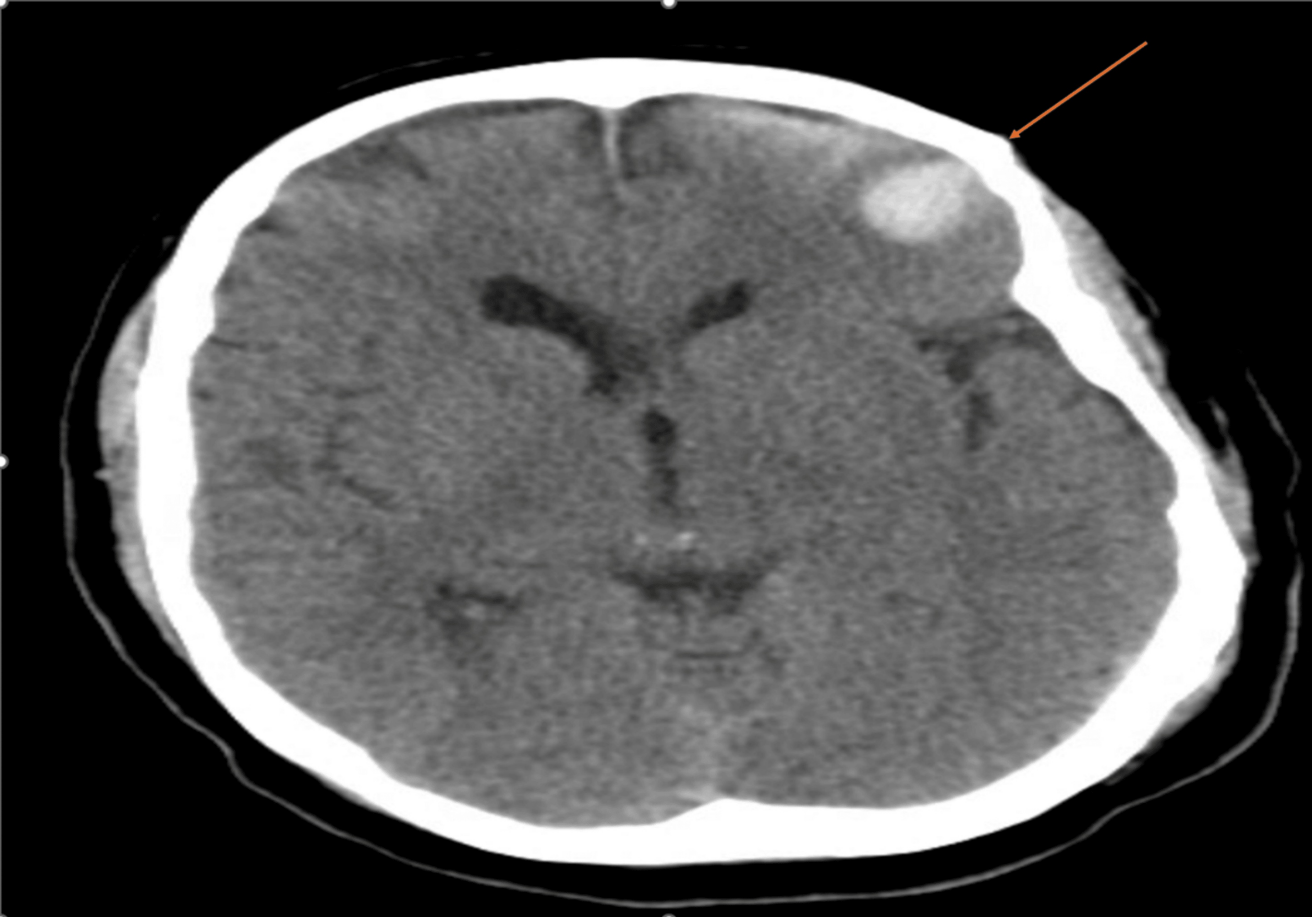
Follow-up CT demonstrating hemorrhage stability (arrow) Arrow demonstrates interval stability and early improvement in traumatic intracranial hemorrhage on follow-up imaging.

A repeat non-contrast CT head obtained approximately 24 hours later (hospital day 1) demonstrated interval worsening of subarachnoid hemorrhage and evolving parenchymal contusion, prompting maintenance of conservative management. Despite interval radiographic progression, the patient remained neurologically intact without clinical deterioration (GCS 15/15, no new focal neurologic deficits). The neurosurgeon consultant recommended continued conservative management with close neurologic monitoring and serial neuroimaging. Conservative management included frequent neurologic checks (every one to two hours) and repeat non-contrast CT head, with cautious escalation of anticoagulation only after radiographic stability and multidisciplinary discussion. A subsequent follow-up non-contrast CT of the head obtained on hospital day 2 demonstrated radiographic stability of the ICH (Figure 3). After radiographic stability was confirmed, heparin was discontinued, and the patient was transitioned to apixaban 5 mg twice daily (without a loading dose), per hematology recommendations. Ultimately, the patient remained neurologically stable without further hemorrhagic progression and was discharged in stable condition with close outpatient follow-up.

Key admission and follow-up laboratory values are summarized in Table 1. 

**Table 1:** Key admission and follow-up laboratory values

Test	Admission	Follow‑up	Reference Range
Hemoglobin (g/dL)	13.6	11.7	13–17
Platelets (×10⁹/L)	140	146	150–400
Creatinine (mg/dL)	1.56	1.30	0.6–1.3
Blood urea nitrogen (mg/dL)	32	18	7–20
Sodium (mmol/L)	139	137	135–145
Bicarbonate/Carbon dioxide (mmol/L)	16	23	22–29
Troponin (ng/L)	54	N/A	<14
Activated partial thromboplastin time (aPTT; seconds)	N/A	>110 → 63.1	23–35

## Discussion

Managing concurrent PE and traumatic ICH presents a complex therapeutic dilemma. Early anticoagulation improves outcomes in PE; however, hemorrhagic progression in ICH can be catastrophic and is often unpredictable [2].

Mechanical thrombectomy allowed rapid reduction of thrombus burden while limiting systemic anticoagulation exposure. This approach is supported by emerging evidence describing catheter-directed mechanical thrombectomy as an option in selected high-risk PE cases when systemic thrombolysis is contraindicated [3].

Serial neuroimaging was essential in guiding management. Our patient initially exhibited early hemorrhage progression, reinforcing the need for caution. Accordingly, anticoagulation was initiated in a staged manner after multidisciplinary discussion and neurosurgical input, balancing the high-risk features of massive PE against the risk of hemorrhagic expansion. Unfractionated heparin was favored initially due to its short half-life and reversibility, allowing rapid cessation if neurologic worsening occurred. After repeated neuroimaging demonstrated radiographic stability without clinical deterioration, transition to oral anticoagulation (apixaban) was pursued for definitive venous thromboembolism treatment. Subsequent radiographic stability enabled carefully staged re-initiation of anticoagulation. Limited observational data and retrospective cohort studies suggest that resumption of anticoagulation as early as 48-72 hours after traumatic ICH may be feasible in carefully selected patients, particularly when repeat neuroimaging demonstrates radiographic stability (i.e., no increase in hemorrhage size, no new hemorrhagic foci, and no worsening mass effect) and the patient remains neurologically stable [4]. In practice, the decision is individualized and should incorporate hematoma characteristics, the need for therapeutic anticoagulation, and multidisciplinary input from neurosurgery and thrombosis teams.

Our experience supports the concept that catheter-based thrombectomy offers a reasonable alternative when systemic thrombolysis poses a high bleeding risk. Likewise, individualized timing of anticoagulation resumption guided by repeat imaging and multidisciplinary input may reduce thrombotic complications while minimizing hemorrhagic progression, including repeat CT head imaging at ~24 hours and again after radiographic stability was achieved.

This case underscores the importance of individualized, multidisciplinary decision-making involving neurosurgery, hematology, interventional radiology, and critical care teams. Continuous reassessment remains essential as thrombotic and hemorrhagic risks evolve over time.

## Conclusions

Concurrent PE and traumatic ICH require careful balancing of thrombotic and hemorrhagic risk. In select cases, staged anticoagulation following radiographic stabilization and multidisciplinary review may allow safe treatment while preventing fatal thromboembolic events. Careful neurologic observation and repeat imaging are essential during this period to promptly detect any hemorrhagic progression. Multidisciplinary collaboration helps ensure that treatment decisions are individualized based on clinical stability and evolving risk. Additional research is needed to provide clearer guidance on anticoagulation timing in this complex setting.

## References

[1] Konstantinides SV, Meyer G, Becattini C (2020). 2019 ESC guidelines for the diagnosis and management of acute pulmonary embolism developed in collaboration with the European Respiratory Society (ERS). Eur Heart J.

[2] Hemphill JC 3rd, Greenberg SM, Anderson CS (2015). Guidelines for the management of spontaneous intracerebral hemorrhage: a guideline for healthcare professionals from the American Heart Association/American Stroke Association. Stroke.

[3] Pandya YK, Tzeng E (2024). Mechanical thrombectomy devices for the management of pulmonary embolism. JVS Vasc Insights.

[4] Jung IH, Yun JH, Kim SJ, Chung J, Lee SK (2023). Anticoagulation and antiplatelet agent resumption timing following traumatic brain injury. Korean J Neurotrauma.

